# Postpartum depressive symptoms and associated factors among women with lactation mastitis: a cross-sectional study

**DOI:** 10.1186/s12884-026-08813-y

**Published:** 2026-03-04

**Authors:** Jianrong Li, Yiming Hou, Nana Chen, Yingyi Fan, Xiaohua Pei

**Affiliations:** 1 Department of Chinese Surgery, Beijing University of Chinese Medicine, Beijing, 100029 China; 2 Department of Breast Surgery, Beijing University of Chinese Medicine Third Affiliated Hospital, Beijing, 100029 China

**Keywords:** Lactation mastitis, Postpartum depressive symptoms, Maternal mental health, Breastfeeding behavior, Emotional distress, Edinburgh postnatal depression scale (EPDS), Cross-sectional study

## Abstract

**Background:**

Lactation mastitis is a common complication during breastfeeding that can negatively impact maternal well-being and breastfeeding continuation. While the physical manifestations of lactation mastitis have been widely studied, its psychological burden and related contributing factors among affected women have received comparatively less attention. This study aimed to investigate postpartum depressive symptoms and associated factors in women diagnosed with lactation mastitis.

**Methods:**

A cross-sectional survey was conducted among 87 women with lactation mastitis at a tertiary hospital in China. Participants completed a structured questionnaire encompassing five domains: demographics and perinatal history, personal and breast history, psychological and emotional state, breastfeeding practices and nipple condition, and infant characteristics and interaction. Postpartum depressive symptoms were assessed using the Edinburgh Postnatal Depression Scale (EPDS). Descriptive statistics, group comparisons, multivariable logistic regression, and sensitivity analyses were used to identify factors associated with elevated EPDS scores.

**Results:**

Overall, 49.4% of participants scored above the clinical threshold for postpartum depressive symptoms on the EPDS. Multivariable logistic regression identified emotional tension during breastfeeding (odds ratio[OR] = 6.807, *P* = 0.016), younger maternal age (OR = 0.794, *P* = 0.023), vaginal delivery (OR = 3.382, *P* = 0.032), and pacifier use (OR = 5.103, *P* = 0.006) as independent correlates of elevated depressive symptoms. These associations remained robust across sensitivity analyses.

**Conclusions:**

Women with lactation mastitis appear to be at heightened risk of postpartum depressive symptoms and are associated with both maternal and infant-related factors. Early identification of psychological distress, combined with integrated breastfeeding and mental health support, may help address maternal needs and enhance postpartum care.

**Supplementary Information:**

The online version contains supplementary material available at 10.1186/s12884-026-08813-y.

## Background

Lactation mastitis is a common and painful condition that affects approximately 3% to 20% of breastfeeding women, most commonly occurring within the first six weeks postpartum [1]. It is characterized by breast swelling, redness, pain, fever, and milk secretion disorders [2]. Mastitis not only disrupts the breastfeeding process but also imposes a substantial psychological burden [3]. Affected women have been reported to experience anxiety, guilt, and frustration due to feeding difficulties, physical discomfort, and a perceived loss of maternal competence [4].

Persistent emotional stress has been associated with the onset of postpartum depression (PPD), one of the most common mental health disorders following childbirth. Breastfeeding has generally been regarded as protective against PPD. However, this relationship is complex and may become a risk factor under certain circumstances, such as when breastfeeding is accompanied by mastitis, latching difficulties, or severe pain during feeding [5]. Recent studies have indicated that women with mastitis may be at increased risk of developing PPD [4, 6].

Prior research has predominantly focused on broad comparisons between breastfeeding and non-breastfeeding women [7], or between individuals with high and low levels of breastfeeding self-efficacy [8, 9]. Such study designs have limited capacity to capture the nuanced psychosocial heterogeneity present within specific clinical populations. Psychological vulnerability in women with mastitis may result not only from the condition itself but also from a range of interacting contextual factors—including emotional tension during breastfeeding, nipple discomfort, infant behavior, pacifier use, and sociodemographic background—which are often overlooked but may shape maternal mental health. However, the combined effects of these factors in women with lactation mastitis remain insufficiently explored.

To address this gap, we employed a multidimensional cross-sectional approach to investigate psychological and breastfeeding-related factors in Chinese women diagnosed with lactation mastitis, focusing on within-group variability to uncover nuanced and modifiable risk factors. The assessment included both conventional indicators of postpartum distress and context-specific measures that reflect emotional tension during breastfeeding and maternal perceptions of infant behavior. By elucidating the psychosocial and physical correlates of depressive symptoms in this population, this study seeks to inform integrated clinical strategies that address both maternal mental health and breastfeeding outcomes.

## Methods

### Study design and participants

This cross-sectional study was conducted at the Breast Department of Beijing University of Chinese Medicine Third Affiliated Hospital. A total of 87 lactating women with a clinical diagnosis of lactation mastitis were consecutively recruited between September 2022 and February 2023. Women aged ≥ 18 years were eligible if they were currently breastfeeding, had received a clinical diagnosis of lactation mastitis, and were capable of providing informed consent. Exclusion criteria included documented cognitive or communication impairments or the inability to independently complete the questionnaire. All participants received information about the study purpose and procedures, and informed consent was obtained before participation. The study protocol was approved by the Ethics Committee of Beijing University of Chinese Medicine Third Affiliated Hospital (BZYSY-2023KYKTPJ-02) and was conducted in accordance with the Declaration of Helsinki.

### Data collection

Data were collected via a structured, self-administered online questionnaire developed using the Wenjuanxing platform (www.wjx.cn; Changsha Ranxing Information Technology Co., Ltd., China). Despite being administered online, all questionnaires were completed under the direct supervision of trained investigators to ensure accuracy and completeness. The questionnaire was developed based on clinical experience, expert consultation, and a focused review of literature on postpartum depression, lactation-related stress, and psychosocial aspects of mastitis. These sources helped identify key thematic areas—such as perinatal risk factors, breastfeeding experience, sleep and fatigue, nipple condition, emotional responses, and infant–mother interaction—which were translated into corresponding items. The questionnaire was subsequently refined after a pilot test involving 20 participants.

The final questionnaire included items covering participant demographic and perinatal information, medical and obstetric history, emotional state during breastfeeding and sleep patterns, breastfeeding practices and nipple condition, and infant characteristics and feeding behaviors (Supplementary file 1). The item assessing emotional state during breastfeeding(defined as a subjective sense of tension or emotional discomfort experienced during breastfeeding, independent of physical pain) was revised to improve clarity in translation, with response options phrased as: *“Yes*,* I feel very relaxed during breastfeeding*,*” “No*,* I do not feel relaxed during breastfeeding*,*” and “I am not currently breastfeeding.”* Given the absence of validated Chinese instruments specifically assessing emotional experiences during breastfeeding, this single-item measure was adopted as a pragmatic way to capture mothers’ subjective feelings during feeding. Although not formally psychometrically validated, it was pilot-tested and demonstrated acceptable clarity and face validity among participants.

The questionnaire was developed following a literature review on postpartum depression, lactation-related stress, and psychosocial aspects of mastitis. Key thematic areas—such as perinatal risk factors, breastfeeding experience, sleep, nipple condition, emotional responses, and infant-mother interaction—were translated into specific items. The study was exploratory, intended to identify potential psychosocial correlates of depressive symptoms.

该问卷是在对产后抑郁症、哺乳相关压力和乳腺炎的社会心理方面的文献综述后制定的。关键主题领域——如围产期危险因素、母乳喂养经历、睡眠、状况、情绪反应和婴儿与母亲的互动——被转化为具体项目。该研究是探索性的, 旨在确定抑郁症状的潜在社会心理相关性。

Postpartum depressive symptoms were measured using the Chinese version of the Edinburgh Postpartum Depression Scale (EPDS). The EPDS is a 10-item self-report instrument originally developed by Cox et al. [10] and later validated by Lee et al. [11] for use in Chinese populations. Each item was rated on a 4-point scale (0–3), yielding a total score ranging from 0 to 30, with higher scores reflecting more severe depressive symptoms. A total score of ≥ 9 has been widely adopted to screen for postpartum depression risk, while a score of ≥ 13 suggests a higher likelihood of clinical depression, consistent with international standards.

Breastfeeding self-efficacy was assessed using the Chinese version of the Breastfeeding Self-Efficacy Scale-Short Form (BSES-SF), a 14-item instrument originally developed by Dennis [12] and culturally adapted for Chinese populations. Each item was rated on a 5-point Likert scale (1–5), producing a total score ranging from 14 to 70, with higher scores reflecting greater confidence in breastfeeding. A total score of ≤ 50 was adopted to indicate low self-efficacy, as supported by prior research identifying this cutoff as a predictor of early breastfeeding cessation [13].

### Data management and statistical analysis

Data were exported from the Wenjuanxing platform and checked for completeness. Descriptive statistics were calculated using SPSS 25.0 (IBM Corp., Armonk, NY, USA), with continuous variables reported as mean ± standard deviation (SD) or medians with interquartile range (IQR), and categorical variables summarized as frequencies and percentages (n [%]). Normality was assessed using the Shapiro-Wilk test. As EPDS and BSES-SF scores were non-normally distributed, non-parametric tests (Kruskal-Wallis or Mann-Whitney U) were applied to continuous variables. Chi-square or Fisher’ s exact tests were applied to categorical variables, as appropriate. Multivariate logistic regression was used to identify factors associated with elevated EPDS scores. Sensitivity analyses were conducted with adjustment for key covariates. Spearman correlation analysis was used to assess associations between continuous variables. Least Absolute Shrinkage and Selection Operator (LASSO) regression was performed using the “glmnet” package in R 4.3.1 (R Foundation, Vienna, Austria), with the optimal penalty parameter (λ) selected via 10-fold cross-validation. All tests were two-sided, with *P* < 0.05 considered statistically significant. Figures were created using GraphPad Prism 10.0 (GraphPad Software, San Diego, CA, USA). No formal sample size calculation was conducted due to the exploratory nature of the study; the sample included all eligible participants recruited during the study period.

## Results

### Participant characteristics

A total of 87 women diagnosed with lactation mastitis were enrolled in the study, all with singleton pregnancies. Some potentially relevant demographic and perinatal information, medical and obstetric history, emotional state during breastfeeding and sleep patterns, breastfeeding practices and nipple condition, and infant characteristics and feeding behaviors are summarized (Table 1). The mean maternal age was 33.0 years (SD = 3.0), and the mean body mass index (BMI) was 22.1 kg/m² (SD = 3.9). The majority of participants held higher education degrees, with 52.9% having a bachelor’s degree, and 89.7% were primiparous. Vaginal delivery accounted for 58.6% of cases, and the majority of pregnancies were naturally conceived.

**Table 1:** Demographic and perinatal information, medical and obstetric history, emotional state during breastfeeding and sleep patterns, breastfeeding practices and nipple condition, and infant characteristics and feeding behaviors of participants

Variable	Value
Demographic and perinatal information	
Age (years)	33.0 ± 3.0
BMI (kg/m²)	22.1 ± 3.9
Highest education level	
Associate degree or below	6 (6.9%)
Bachelor’s degree	46 (52.9%)
Postgraduate or higher	35 (40.2%)
Primiparous (first birth)	78 (89.7%)
Planned pregnancy	77 (88.5%)
Conception method	
Natural conception	78 (89.7%)
Assisted reproductive technology (ART)	9 (10.3%)
Mode of delivery	
Vaginal (including assisted)	51 (58.6%)
Cesarean section	36 (41.4%)
Medical and obstetric history	
Previous lactation mastitis	59 (67.8%)
Chronic illness history	20 (23.0%)
Breast surgery history	3 (3.4%)
Family history of breast cancer	4 (4.6%)
Allergy (drug or food)	8 (9.2%)
Smoker	3 (3.4%)
Alcohol use	10 (11.5%)
Emotional state during breastfeeding and sleep patterns	
EPDS score (0–30)	8.7 ± 6.2
EPDS < 9	44 (50.6%)
EPDS 9–12	19 (21.8%)
EPDS ≥ 13	24 (27.6%)
BSES-SF score (14–70)	49.5 ± 14.6
BSES-SF < 50	42 (48.3%)
BSES-SF ≥ 50	45 (51.7%)
Emotional state during breastfeeding	
Relaxed	69 (79.3%)
Tense	15 (17.2%)
Not breastfeeding	3 (3.4%)
Nighttime sleep duration (hours)	5.7 ± 1.4
Breastfeeding practices and nipple condition	
Exclusive breastfeeding	42 (48.3%)
On-demand feeding	65 (74.7%)
Pacifier use	30 (34.5%)
Breast pump use	
Daily	51 (58.6%)
Occasional	32 (36.8%)
None	4 (4.6%)
Poor latch (infant only on nipple)	29 (33.3%)
Nipple Abnormalities	
Damaged nipples	55 (63.2%)
Inverted nipples	14 (16.1%)
Flat nipples	34 (39.1%)
Infant characteristics and feeding behaviors	
Infant age at mastitis onset (months)	5.4 ± 4.4
Preterm birth (< 37 weeks)	6 (7.1%)
Neonatal jaundice	28 (32.2%)
Infant colic (gassiness)	39 (44.8%)
Infant breast refusal	13 (14.9%)
Infant easily distracted during feeding	40 (46.0%)

Data are presented as mean ± SD for continuous variables and as n (%) for categorical variables. Percentages are calculated based on the total number of participants within each column

In terms of medical and breast-related history, 67.8% of participants had a history of lactation mastitis. A smaller proportion reported chronic illness (23.0%), prior breast surgery (3.4%), or a family history of breast cancer (4.6%). Smoking and alcohol use were rare, reported at 3.4% and 11.5%, respectively.

**Fig. 1:**
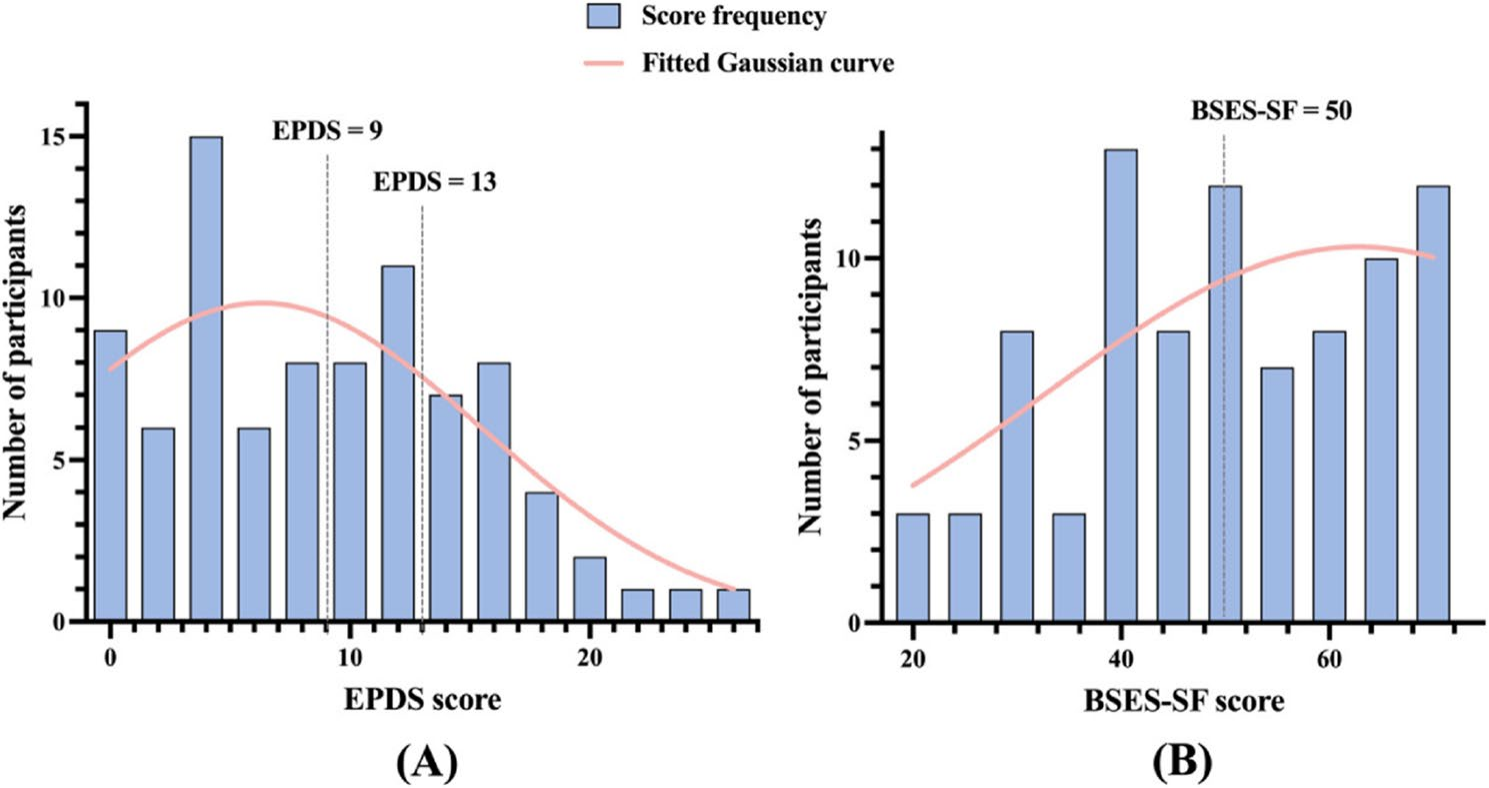
Distribution of psychological assessment scores among women with lactation mastitis (**A**) EPDS scores (**B**) BSES-SF scores. Note. **A** The histogram shows the number of participants across EPDS score bins. The smoothed curve represents the overall distribution trend. Dashed lines indicate the established cutoff scores of 9 and 13, which define thresholds for possible and probable postpartum depression. **B **The histogram illustrates the distribution of BSES-SF scores. Dashed line at 50 represents the widely used cutoff to distinguish between low and high breastfeeding self-efficacy

Psychological assessment showed that 21.8% of participants scored between 9 and 12 on the EPDS, and 27.6% scored ≥ 13, indicating elevated depressive symptoms in a substantial proportion of the sample. The mean EPDS score was 8.7 (SD = 6.2). Breastfeeding self-efficacy, as measured by the BSES-SF, had a mean score of 49.5 (SD = 14.6), with 48.3% of participants scoring below the 50-point threshold. While 79.3% of mothers reported feeling relaxed during breastfeeding, 17.2% self-reported experiencing emotional tension. EPDS and BSES-SF scores were stratified based on established clinical thresholds to illustrate participants’ psychological status (Fig. 1A-B).

### Comparison of maternal and infant characteristics across EPDS and BSES-SF groups

**Fig. 2:**
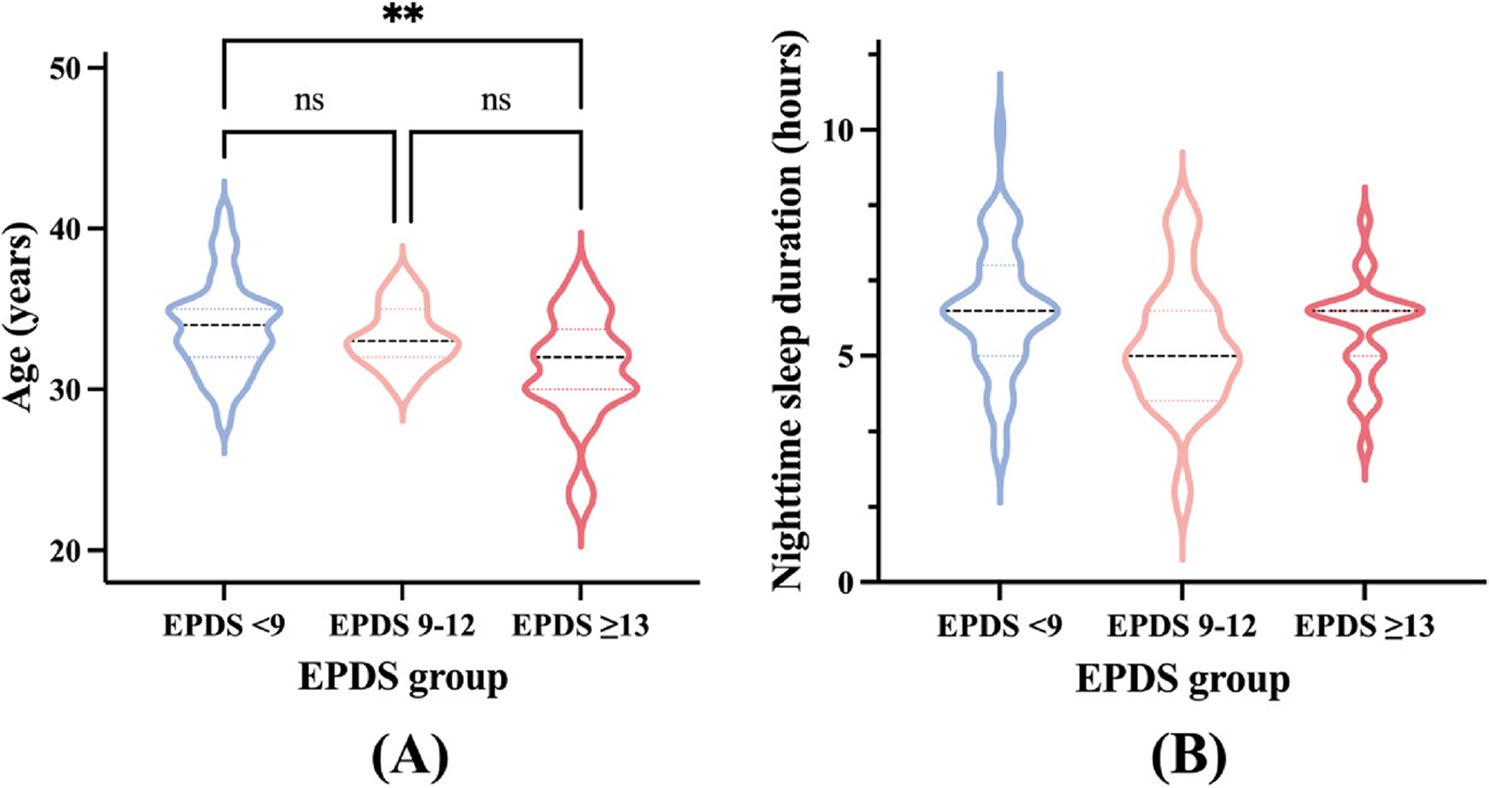
Group differences in (**A**) maternal age and (**B**) nighttime sleep duration across three EPDS score categories. Note.Violin plots show distribution and group comparisons. *P < 0.05, **P < 0.01

**Fig. 3:**
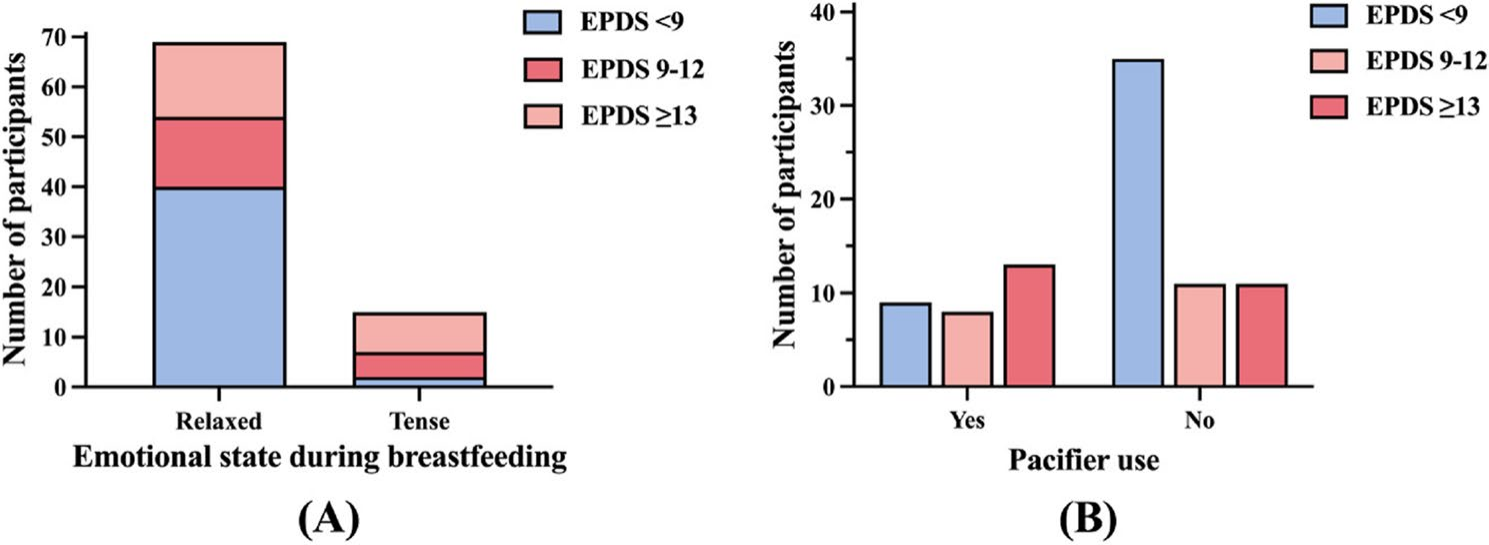
Emotional tension (**A**) and pacifier use (**B**) by EPDS score category. Note. Bar charts represent proportion of responses

Based on EPDS scores, participants were classified into three groups: <9 (*n* = 44), 9–12 (*n* = 19), and ≥ 13 (*n* = 24). Participants with higher EPDS scores were more likely to be younger, to report emotional tension during breastfeeding, and to report pacifier use, with all comparisons reaching statistical significance (Table 2). A non-significant trend toward shorter nighttime sleep duration was also observed among participants with higher EPDS scores. These associations were further illustrated using violin plots and bar charts to highlight the distributional differences across EPDS groups (Figs. 2A-B and 3A-B).

**Table 2:** Comparison of maternal and infant characteristics by EPDS score category

Variable	EPDS < 9 (*n* = 44)	EPDS 9–12 (*n* = 19)	EPDS ≥ 13 (*n* = 24)	Test statistic
Continuous variables				
Age (years)	34.0 (32.0, 35.0)	33.0 (32.0, 35.0)	32.0 (30.0, 33.8)	*H* = 9.055, *P* = 0.011*
BMI (kg/m²)	22.0 (19.3, 24.1)	21.2 (18.4, 23.0)	21.6 (19.7, 24.1)	*H* = 1.725, *P* = 0.422
BSES-SF score	53.0 (40.3, 64.0)	51.0 (39.0, 59.0)	43.5 (31.8, 56.3)	*H* = 2.900, *P* = 0.235
Nighttime sleep duration (hours)	6.0 (5.0,7.0)	5.0 (4.0, 6.0)	6.0 (5.0, 6.0)	*H* = 4.234, *P* = 0.120
Infant age at mastitis onset (months)	4.3(1.8, 8.8)	3.200(1.2, 7.1)	4.6 (2.1, 7.7)	*H* = 1.411, *P* = 0.494
Categorical variables				
Highest education level				χ2 = 3.754, *P* = 0.44
Associate degree or below	4(9.1%)	0(0.0%)	2(8.3%)	
Bachelor’ s degree	21(47.7%)	12(63.2%)	13(54.2%)	
Postgraduate or higher	19(43.2%)	7(36.8%)	9(37.5%)	
Planned pregnancy				χ2 = 0.042, *P* = 0.979
Yes	39(88.6%)	17(89.5%)	21(87.5%)	
No	5(11.4%)	2(10.5%)	3(12.5%)	
Conception method				χ2 = 1.72, *P* = 0.423
Natural conception	38(86.4%)	17(89.5%)	23(95.8%)	
ART	6(13.6%)	2(10.5%)	1(4.2%)	
Primiparous (first birth)				χ2 = 1.217, *P* = 0.544
Yes	38(86.4%)	18(94.7%)	22(91.7%)	
No	6(13.6%)	1(5.3%)	2(8.3%)	
Mode of delivery				χ2 = 5.538, *P* = 0.063
Vaginal (including assisted)	21(47.7%)	15(79.0%)	15(62.5%)	
Cesarean section	23(52.3%)	4(21.1%)	9(37.5%)	
Previous lactation mastitis				χ2 = 4.83, *P* = 0.089
Yes	33(75.0%)	14(73.7%)	12(50.0%)	
No	11(25.0%)	5(26.3%)	12(50.0%)	
Chronic illness history				χ2 = 5.072, *P* = 0.079
Yes	11(25.0%)	7(36.8%)	2(8.3%)	
No	33(75.0%)	12(63.2%)	22(91.7%)	
Breast surgery history				χ2 = 1.992, *P* = 0.369
Yes	2(4.6%)	1(5.3%)	0(0.0%)	
No	42(95.5%)	18(94.7%)	24(100.0%)	
Family history of breast cancer				χ2 = 3.391, *P* = 0.183
Yes	2(4.6%)	2(10.5%)	0(0.0%)	
No	42(95.5%)	17(89.5%)	24(100.0%)	
Allergy (drug or food)				χ2 = 5.694, *P* = 0.058
Yes	5(11.3%)	3(15.8%)	0(0.0%)	
No	39(88.6%)	16(84.2%)	24(100.0%)	
Smoker				χ2 = 4.496, *P* = 0.106
Yes	0(0.0%)	1(5.3%)	2(8.3%)	
No	44(100.0%)	18(94.7%)	22(91.7%)	
Alcohol use				χ2 = 2.816, *P* = 0.245
Yes	3(6.8%)	2(10.5%)	5(20.8%)	
No	41(93.2%)	17(89.5%)	19(79.2%)	
Emotional state during breastfeeding				χ2 = 12.64, *P* = 0.013*
Relaxed	40(90.9%)	14(73.7%)	15(62.5%)	
Tense	2(4.6%)	5(26.3%)	8(33.3%)	
Not breastfeeding	2(4.6%)	0(0.0%)	1(4.2%)	
Exclusive breastfeeding				χ2 = 1.129, *P* = 0.569
Yes	21(47.7%)	11(57.9%)	10(41.7%)	
No	23(52.3%)	8(42.1%)	14(58.3%)	
On-demand feeding				χ2 = 1.377, *P* = 0.502
Yes	35(79.6%)	14(73.7%)	16(66.7%)	
No	9(20.5%)	5(26.3%)	8(33.3%)	
Pacifier use				χ2 = 8.437, *P* = 0.015*
Yes	9(20.5%)	8(42.1%)	13(54.2%)	
No	35(79.6%)	11(57.9%)	11(45.8%)	
Breast pump use				χ2 = 1.679, *P* = 0.795
Daily	23(52.3%)	12(63.2%)	16(66.7%)	
Occasional	19(43.2%)	6(31.6%)	7(29.2%)	
None	2(4.6%)	1(5.3%)	1(4.2%)	
Poor latch (infant only on nipple)				χ2 = 3.96, *P* = 0.138
Yes	19(43.2%)	4(21.1%)	6(25.0%)	
No	25(56.8%)	15(79.0%)	18(75.0%)	
Inverted nipples				χ2 = 1.49, *P* = 0.475
Yes	5(11.4%)	4(21.1%)	5(20.8%)	
No	39(88.6%)	15(79.0%)	19(79.2%)	
Flat nipples				χ2 = 0.277, *P* = 0.871
Yes	16(36.4%)	8(42.1%)	10(41.7%)	
No	28(63.6%)	11(57.9%)	14(58.3%)	
Damaged nipples				χ2 = 1.744, *P* = 0.418
Yes	28(63.6%)	14(73.7%)	13(54.2%)	
No	16(36.4%)	5(26.3%)	11(45.8%)	
Preterm birth (< 37 weeks)				χ2 = 3.434, *P* = 0.488
Yes	3(7.1%)	2(10.5%)	1(4.2%)	
No	39(92.9%)	17(89.5%)	23(95.8%)	
(Excluded *n* = 2 not shown)				
Neonatal jaundice				χ2 = 1.478, *P* = 0.478
Yes	12(27.3%)	6(31.6%)	10(41.7%)	
No	32(72.7%)	13(68.4%)	14(58.3%)	
Infant colic (gassiness)				χ2 = 1.409, *P* = 0.494
Yes	17(38.6%)	10(52.6%)	12(50.0%)	
No	27(61.4%)	9(47.4%)	12(50.0%)	
Infant breast refusal				χ2 = 2.442, *P* = 0.295
Yes	5(11.4%)	2(10.5%)	6(25.0%)	
No	39(88.6%)	17(89.5%)	18(75.0%)	
Infant easily distracted during feeding				χ2 = 5.729, *P* = 0.057
Yes	17(38.6%)	7(36.8%)	16(66.7%)	
No	27(61.4%)	12(63.2%)	8(33.3%)	

Data are presented as median (IQR) for continuous variables and n (%) for categorical variables. Percentages are calculated based on the total number of participants within each column. Group comparisons were conducted using the Kruskal–Wallis test (H statistic) for continuous variables, and the chi-square test for categorical variables

* *P* < 0.05, ** *P* < 0.01

Participants were grouped according to BSES-SF scores (< 50 = low, ≥ 50 = high) to assess differences in breastfeeding self-efficacy. Compared with participants in the low self-efficacy group, those with high self-efficacy exhibited a significantly later onset of mastitis (*P* < 0.001), were more likely to report feeling relaxed during breastfeeding (*P* < 0.001), and showed higher rates of exclusive breastfeeding (*P* < 0.001) and on-demand feeding (*P* = 0.031). Additionally, participants in the low self-efficacy group reported higher rates of breastfeeding-related difficulties and infant feeding problems, including poor latch (*P* < 0.001), flat nipples (*P* = 0.044), neonatal jaundice (*p* = 0.040), and infant breast refusal (*P* < 0.001) (Table 3).

**Table 3:** Comparison of maternal and infant characteristics by BSES-SF score category

Variable	BSES-SF < 50 (*n* = 42)	BSES-SF ≥ 50 (*n* = 45)	Test statistic
Continuous variables			
Age (years)	33.0 (31.0, 35.0)	33.0 (31.0, 35.0)	*z* = −0.171, *P* = 0.864
BMI (kg/m²)	21.3 (19.6, 24.2)	21.8 (18.6, 23.9)	*z* = −0.178, *P* = 0.858
EPDS score	10.5 (4.0, 13.3)	8.0 (3.0, 11.5)	*z* = −1.145, *P* = 0.252
Nighttime sleep duration (hours)	6.0 (5.0, 6.0)	6.0 (5.0, 7.0)	*z* = −1.710, *P* = 0.087
Infant age at mastitis onset (months)	2.7 (1.4, 4.5)	6.8 (2.8, 9.9)	*z* = −3.857, *P* = 0.000**
Categorical variables			
Highest education level			χ2 = 1.293, *P* = 0.524
Associate degree or below	4(9.5%)	2(4.4%)	
Bachelor’ s degree	23(54.8%)	23(51.1%)	
Postgraduate or higher	15(35.7%)	20(44.4%)	
Planned pregnancy			*P* = 0.512
Yes	36(85.7%)	41(91.1%)	
No	6(14.3%)	4(8.9%)	
Conception method			*P* = 0.733
Natural conception	37(88.1%)	41(91.1%)	
ART	5(11.9%)	4(8.9%)	
Primiparous (first birth)			*P* = 1.000
Yes	38(90.5%)	40(88.9%)	
No	4(9.5%)	5(11.1%)	
Mode of delivery			χ2 = 0.073, *P* = 0.787
Vaginal (including assisted)	24(57.1%)	27(60.0%)	
Cesarean section	18(42.9%)	18(40.0%)	
Previous lactation mastitis			χ2 = 6.340, *P* = 0.012*
Yes	23(54.8%)	36(80.0%)	
No	19(45.2%)	9(20.0%)	
Chronic illness history			χ2 = 0.112, *P* = 0.738
Yes	9(21.4%)	11(24.4%)	
No	33(78.6%)	34(75.6%)	
Breast surgery history			*P* = 1.000
Yes	1(2.4%)	2(4.4%)	
No	41(97.6%)	43(95.6%)	
Family history of breast cancer			*P* = 1.000
Yes	2(4.8%)	2(4.4%)	
No	40(95.2%)	43(95.6%)	
Allergy (drug or food)			*P* = 1.000
Yes	4(9.5%)	4(8.9%)	
No	38(90.5%)	41(91.1%)	
Smoker			*P* = 0.608
Yes	2(4.8%)	1(2.2%)	
No	40(95.2%)	44(97.8%)	
Alcohol use			*P* = 0.512
Yes	6(14.3%)	4(8.9%)	
No	36(85.7%)	41(91.1%)	
Emotional state during breastfeeding			χ2 = 17.301, *P* = 0.000**
Relaxed	26(61.9%)	43(95.6%)	
Tense	13(31.0%)	2(4.4%)	
Not breastfeeding	3(7.1%)	0(0.0%)	
Exclusive breastfeeding			χ2 = 19.466, *P* = 0.000**
Yes	10(23.8%)	32(71.1%)	
No	32(76.2%)	13(28.9%)	
On-demand feeding			χ2 = 4.673, *P* = 0.031*
Yes	27(64.3%)	38(84.4%)	
No	15(35.7%)	7(15.6%)	
Pacifier use			χ2 = 1.291, *P* = 0.256
Yes	17(40.5%)	13(28.9%)	
No	25(59.5%)	32(71.1%)	
Breast pump use			χ2 = 3.928, *P* = 0.140
Daily	29(69.1%)	22(48.9%)	
Occasional	12(28.6%)	20(44.4%)	
None	1(2.4%)	3(6.7%)	
Poor latch (infant only on nipple)			χ2 = 13.257, *P* = 0.000**
Yes	6(14.3%)	23(51.1%)	
No	36(85.7%)	22(48.9%)	
Inverted nipples			χ2 = 3.582, *P* = 0.058
Yes	10(23.8%)	4(8.9%)	
No	32(76.2%)	41(91.1%)	
Flat nipples			χ2 = 4.067, *P* = 0.044*
Yes	21(50.0%)	13(28.9%)	
No	21(50.0%)	32(71.1%)	
Damaged nipples			χ2 = 1.187, *P* = 0.276
Yes	29(69.1%)	26(57.8%)	
No	13(31.0%)	19(42.2%)	
Preterm birth (< 37 weeks)			χ2 = 2.682, *P* = 0.262
Yes	3(7.1%)	3(7.0%)	
No	39(92.9%)	40(93.0%)	
(Excluded *n* = 2 not shown)			
Neonatal jaundice			χ2 = 4.238, *P* = 0.040*
Yes	18(42.9%)	10(22.2%)	
No	24(57.1%)	35(77.8%)	
Infant colic (gassiness)			χ2 = 0.878, *P* = 0.349
Yes	21(50.0%)	18(40.0%)	
No	21(50.0%)	27(60.0%)	
Infant breast refusal			χ2 = 16.375, *P* = 0.000**
Yes	13(31.0%)	0(0.0%)	
No	29(69.1%)	45(100.0%)	
Infant easily distracted during feeding			χ2 = 2.523, *P* = 0.112
Yes	23(54.8%)	17(37.8%)	
No	19(45.2%)	28(62.2%)	

Continuous variables are presented as median (IQR) and compared using the Mann-Whitney U test. Categorical variables are presented as n (%), with percentages calculated based on the total number of participants within each column, and compared using the chi-square test or Fisher’ s exact test, as appropriate

*P < 0.05, **P < 0.01

### logistic regression and sensitivity analysis

Among the two psychological indicators assessed, EPDS scores were selected as the primary outcome variable because of their clinical relevance and direct representation of depressive symptom severity. To explore potential contributing factors, a multivariable logistic regression model was constructed. Covariates included maternal age, delivery mode, history of mastitis, pacifier use, and additional variables identified as significant in bivariate analyses. Selection of these covariates was based on both clinical relevance and statistical criteria (Table 4). For ease of interpretation and modeling, EPDS scores were dichotomized using a clinically validated cutoff of 9.

**Table 4:** Multivariable logistic regression model for elevated EPDS scores (≥ 9)

Variable	OR (95% CI)	*P*-value
Age	0.794 (0.650–0.968)	0.023*
Mode of delivery	3.382 (1.109–10.313)	0.032*
Previous lactation mastitis	0.645 (0.205–2.036)	0.455
Allergy (drug or food)	0.319 (0.055–1.838)	0.201
Emotional state during breastfeeding	6.807 (1.428–32.452)	0.016*
Pacifier use	5.103 (1.601–16.263)	0.006**
Infant easily distracted during feeding	1.755 (0.604–5.101)	0.302

ORs and 95% confidence intervals (CIs) are reported

*P < 0.05, **P < 0.01

**Fig. 4:**
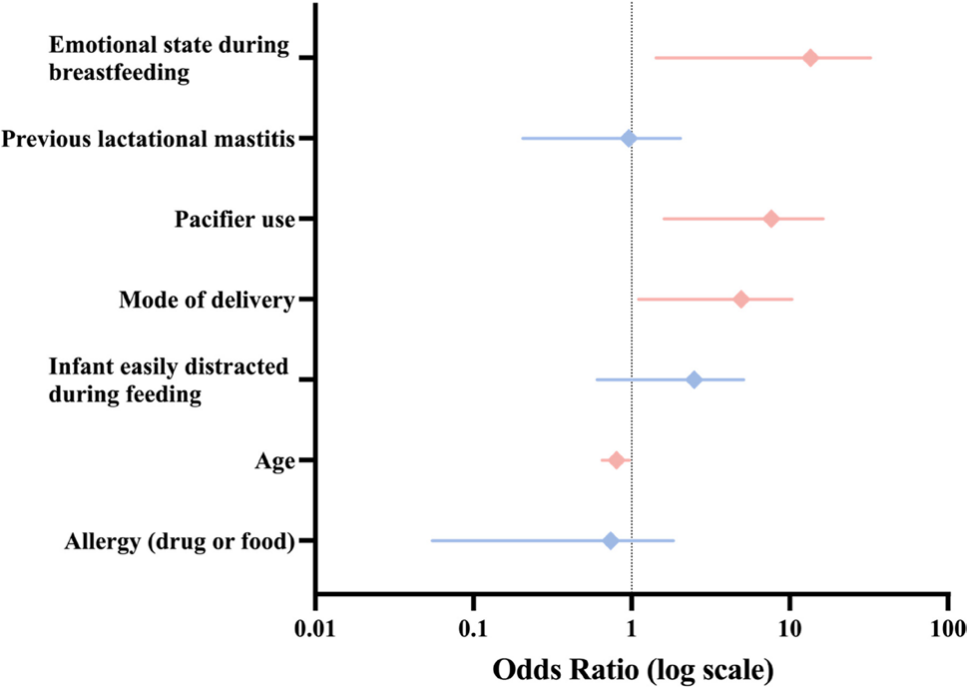
Forest plot of adjusted ORs from the multivariable logistic regression model for elevated EPDS scores (≥ 9). Note. ORs and 95% CIs are reported

Younger maternal age was significantly associated with increased odds of elevated depressive symptoms (odds ratio[OR] = 0.794, *P* = 0.023). Moreover, vaginal delivery (OR = 3.382, *P* = 0.032), pacifier use (OR = 5.103, *P* = 0.006), and emotional tension during breastfeeding (OR = 6.807, *P* = 0.016) emerged as independent risk factors for elevated EPDS scores (Table 4; Fig. 4). The model demonstrated moderate explanatory power, with a Nagelkerke R² of 0.423 and a McFadden R² of 0.276.

**Fig. 5:**
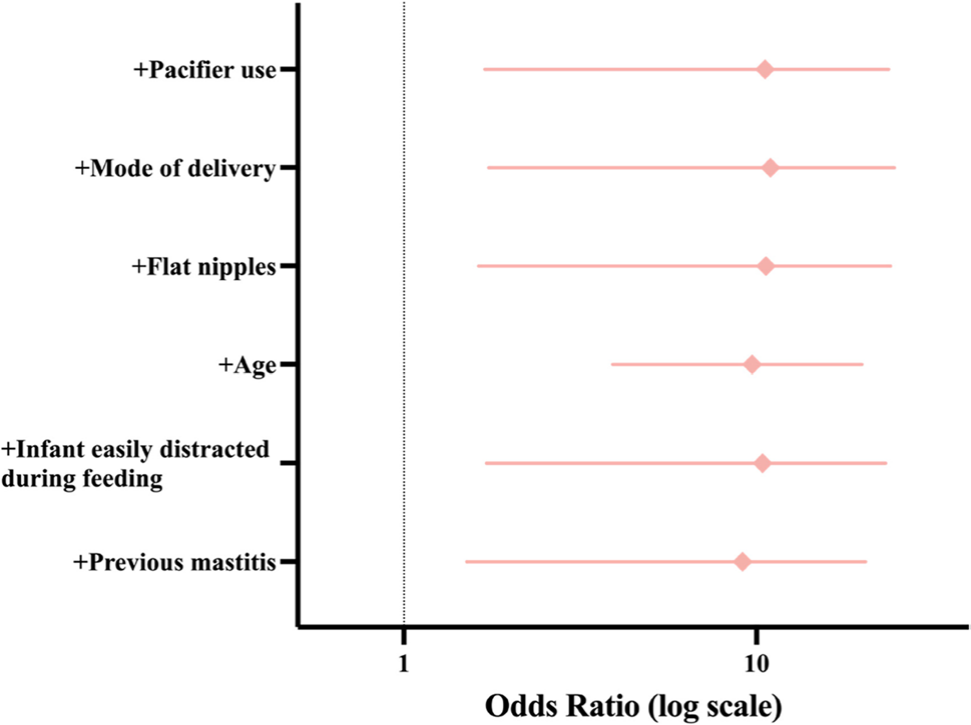
Sensitivity analysis: forest plots of ORs for emotional tension adjusted for single covariates. Note. ORs and 95% CIs are presented

To further evaluate the robustness of the association between emotional tension during breastfeeding and postpartum depressive symptoms, a series of two-variable logistic regression models was conducted. Emotional tension was retained as the primary predictor, while each covariate was introduced separately. This variable was selected based on its high odds ratio and statistical significance in the multivariable model. The association between emotional tension and elevated EPDS scores remained statistically significant across all models (Table 5; Fig. 5), reinforcing its potential as a robust indicator of psychological vulnerability. Additionally, several covariates—including younger maternal age, vaginal delivery, and pacifier use—also demonstrated consistent statistical significance in their respective models. The reproducibility of these results across both adjusted and minimally adjusted models supports the role of these factors as independent contributors to postpartum depressive symptoms.

**Table 5:** Sensitivity analysis using two-variable logistic regression models

Variable	OR (95% CI)	*P*-value
Group 1		
Emotional state during breastfeeding	5.350 (1.440–19.885)	0.012*
Age	0.826 (0.697–0.979)	0.028*
Group 2		
Emotional state during breastfeeding	6.543 (1.741–24.593)	0.005**
Mode of delivery	2.843 (1.106 ~ 7.308)	0.030*
Group 3		
Emotional state during breastfeeding	5.542 (1.509–20.357)	0.010*
Previous mastitis	0.616 (0.235–1.615)	0.324
Group 4		
Emotional state during breastfeeding	6.340 (1.696–23.701)	0.006**
Pacifier use	4.075 (1.476–11.249)	0.007*
Group 5		
Emotional state during breastfeeding	6.254 (1.629–24.000)	0.008**
Flat nipples	0.870 (0.329–2.298)	0.779
Group 6		
Emotional state during breastfeeding	6.308 (1.714–23.210)	0.006**
Infant easily distracted during feeding	2.035 (0.820–5.049)	0.125

ORs and 95% CIs are reported

*P < 0.05, **P < 0.01

### LASSO regression analysis

To complement the multivariable logistic regression and mitigate potential overfitting, a penalized logistic regression was conducted using the LASSO. The binary outcome variable was defined as elevated depressive symptoms (EPDS ≥ 9) and a total of twenty maternal, behavioral, and infant-related variables were included as candidate predictors. The LASSO model identified five variables with non-zero coefficients. Emotional tension during breastfeeding was retained in both the multivariable and LASSO models, reinforcing its role as a robust and consistent predictor. Additionally, short nighttime sleep (< 5 h), smoking history, previous breast surgery, and flat nipples were selected by the LASSO model, suggesting potential associations that warrant further investigation (Supplementary Table S1).

### Correlation analysis

**Fig. 6:**
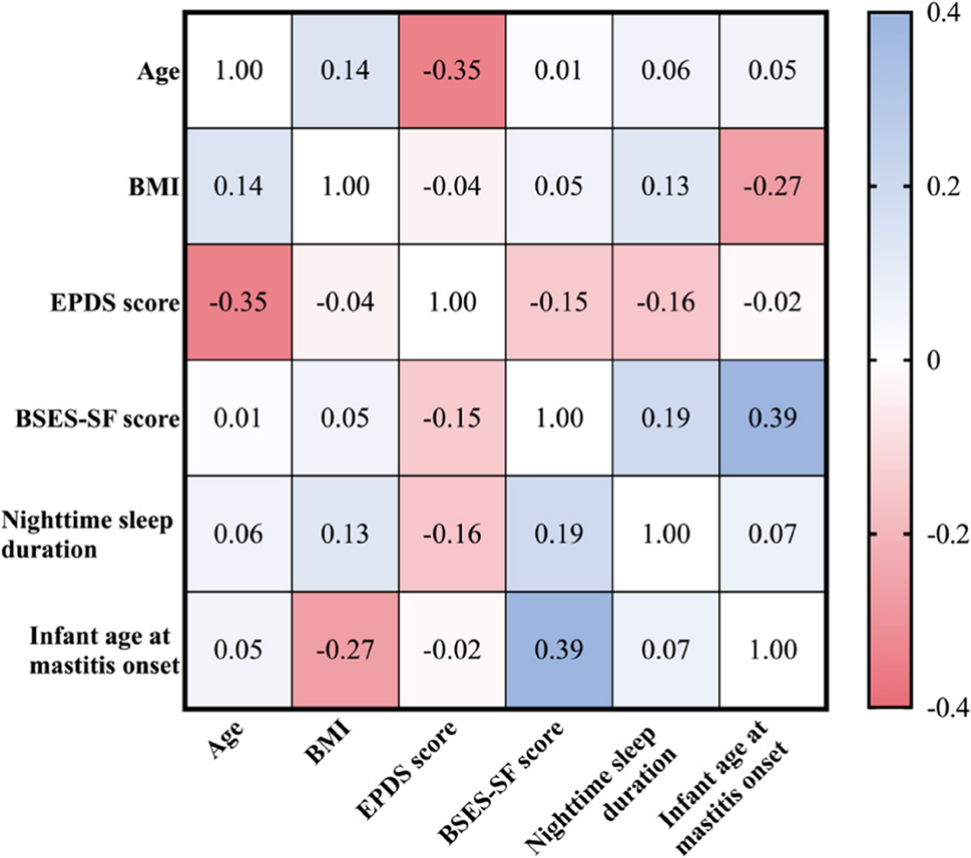
Spearman correlation heatmap of selected continuous variables. Notes. The heatmap displays pairwise Spearman correlation coefficients (ρ) among six key continuous variables. Color intensity reflects the strength and direction of correlation coefficients

To further examine the interrelationships among key psychological, behavioral, and infant-related factors, Spearman’s rank correlation analysis was performed using selected continuous variables (Fig. 6). EPDS scores exhibited a moderate negative correlation with maternal age (ρ = −0.35), indicating increased susceptibility to depressive symptoms among younger mothers. Breastfeeding self-efficacy scores showed a moderate positive correlation with infant age at the onset of mastitis (ρ = 0.39), suggesting that higher self-efficacy may be associated with delayed onset of lactation mastitis.

## Discussion

Although lactation mastitis has been associated with psychological distress, few studies have systematically explored the emotional and behavioral correlates of postpartum depression within this clinical subgroup. In this study, depressive symptoms were examined in relation to maternal-infant characteristics in a sample of 87 affected women. The results reveal complex interactions among maternal, infant, and dyadic factors, and highlight the multifaceted psychological burden experienced by women with mastitis. These findings contribute to a broader understanding of how specific breastfeeding-related stressors may influence postpartum mood disturbances in this population.

Among women diagnosed with lactation mastitis in this study, nearly half (49.4%) scored ≥ 9 on the EPDS, and 27.6% met the threshold for probable depression (EPDS ≥ 13). A recent meta-analysis estimated the global prevalence of postpartum depression at approximately 17.7%; yet these rates were substantially higher, underscoring the psychological vulnerability of this population [14]. These findings reinforce the notion that mastitis is not merely a physiological complication, but also functions as a psychological stressor that warrants clinical attention. Pain has been shown to exhibit a bidirectional relationship with depression [15], and severe acute postpartum pain has been significantly associated with increased risk of postpartum depression [16, 17]. The experience of physical discomfort, combined with frustration due to disrupted breastfeeding and concerns about insufficient milk supply, may contribute to emotional distress, self-doubt, and maternal role failure [5, 6]. Moreover, the social stigma surrounding breastfeeding challenges may discourage timely help-seeking, potentially exacerbating emotional isolation [18]. While mastitis has traditionally been understood as an infectious or mechanical complication of lactation, the current findings support its emerging recognition as a high-risk context for maternal psychological distress. Incorporating routine psychological screening and emotional support into lactation care may represent a key component of comprehensive postpartum health strategies.

A key finding of this study was the robust association between maternal emotional tension during direct breastfeeding and elevated depressive symptoms. After adjustment for multiple maternal and infant covariates, emotional tension during breastfeeding remained an independent predictor of higher EPDS scores, underscoring its potential role as a proximal indicator of maternal psychological distress. While prior research has primarily focused on distal or general predictors of postpartum depression—such as socioeconomic status [19], family support, or antenatal mental health [20]—limited attention has been given to the emotional dimension of the breastfeeding experience itself. Emotional responses occurring in real time during breastfeeding may reflect underlying vulnerability to depression or contribute to symptom exacerbation through a reciprocal feedback mechanism. This observation is clinically meaningful and novel, supporting the value of integrating emotional tension assessments during breastfeeding—such as self-reported tension, irritability, or nervousness—into routine postpartum depression screening protocols. These real-time emotional indicators may provide immediate, contextually relevant cues for early identification and timely intervention among mothers at elevated risk.

Several maternal and infant characteristics were associated with postpartum depressive symptoms in this study, highlighting additional contextual and intervention targets. Younger maternal age was significantly associated with higher EPDS scores. This may result from the compounding psychological stress of managing both mastitis and newborn care, which can be particularly burdensome for younger mothers. A global survey of more than 1.1 million new mothers reported that the highest prevalence of self-reported postpartum depression occurred among those aged 18–24, with incidence decreasing steadily with age [21]. Previous studies have shown that younger mothers are more likely to face psychological challenges such as social isolation, single parenting, family conflict, low self-esteem, first-time childbirth, and body image dissatisfaction, all of which may elevate the risk of postpartum depression [22]. These findings emphasize the importance of routine mental health screening and targeted support strategies for young mothers during the breastfeeding period.

Vaginal delivery was positively associated with elevated depressive symptoms in this study. It is traditionally believed that cesarean sections involve prolonged recovery, greater complication rates, and a higher risk of postpartum depression. However, the psychological experience of childbirth—rather than the delivery mode itself—plays a crucial role in maternal mental well-being [23]. Vaginal deliveries involving obstetric interventions or a perceived lack of control have been linked to increased risk of postpartum emotional distress, particularly among primiparous women [24]. Thus, the findings may reflect not the mode of delivery per se, but rather adverse subjective experiences during labor, which may be more prevalent or perceived as distressing in the context of vaginal birth. These results underscore the importance of assessing not only clinical delivery outcomes but also maternal emotional experiences during labor when evaluating postpartum psychological risk.

Pacifier use was significantly associated with elevated postpartum depressive symptoms (*P* < 0.01 in logistic regression). Rather than serving as a causal factor, pacifier use may reflect underlying maternal emotional distress or breastfeeding difficulties. Mothers coping with an unsettled infant or painful breastfeeding—both common in mastitis—may rely on pacifiers as a compensatory strategy. This finding aligns with previous evidence linking pacifier use to shorter durations of exclusive breastfeeding [25]. Although some studies suggest pacifiers may relieve stress and promote continued breastfeeding among high-risk mothers [26], the present findings suggest that pacifier use in this context is more likely a marker of maternal vulnerability than a protective tool. Clinically, these findings underscore the importance of examining the underlying motivations for pacifier use, as it may signal unmet needs related to breastfeeding support or maternal mental health.

The LASSO analysis further highlighted several variables that may be associated with depressive symptoms, including short nighttime sleep, smoking history, and flat nipples. Although not statistically significant in the primary regression, their inclusion suggests possible relevance and warrants further investigation.

This study has several limitations. Its cross-sectional design precludes causal inference, and the limited, single-center sample may reduce external validity. Only 15 participants in our sample reported emotional tension during breastfeeding, which may limit the representativeness of this subgroup and increase the risk of type I error. While major confounders were adjusted for, residual bias cannot be entirely ruled out, and prior mental health status was not assessed. The self-developed questionnaire used in this study was not psychometrically validated, and its item grouping was pragmatically defined for clinical data collection purposes. In particular, the single-item measure of emotional tension during breastfeeding may be subject to measurement bias and warrants future psychometric evaluation. Nevertheless, the findings provide valuable insights that merit replication in larger, diverse populations to better understand the temporal dynamics between maternal emotional tension during breastfeeding and postpartum depression, particularly among women with lactation mastitis.

## Conclusions

This study underscores the multidimensional relationships between maternal psychological status, breastfeeding behavior, and infant-related factors among women diagnosed with lactation mastitis. Emotional tension during breastfeeding was independently associated with postpartum depressive symptoms. Younger maternal age, vaginal delivery, and pacifier use were also associated with increased depressive symptoms, offering contextual insight into maternal emotional vulnerability. These results emphasize the importance of early psychosocial screening and individualized breastfeeding support for mothers with mastitis. Integrating mental health assessments into breastfeeding consultations may help identify at-risk mothers and provide opportunities for timely support.

## Supplementary Information

Supplementary Material 1.

Supplementary Material 2.

## Data Availability

The datasets generated and analysed during the current study are not publicly available due to concerns regarding participant privacy, but are available from the corresponding author on reasonable request.

## Abbreviations

EPDS: Edinburgh Postnatal Depression Scale
PPD: Postpartum depression
BSES-SF: Breastfeeding Self-Efficacy Scale-Short Form
SD: Standard deviation
IQR: Interquartile range
LASSO: Least Absolute Shrinkage and Selection Operator
BMI: Body mass index
ART: Assisted reproductive technology
OR: Odds ratio
CI: Confidence interval
