# Supplementary material for: Postpartum depressive symptoms and associated factors among women with lactation mastitis: a cross-sectional study
Source: BMC Pregnancy Childbirth. 2026 Mar 4;26:394. doi: 10.1186/s12884-026-08813-y (PMC13067529; doi:10.1186/s12884-026-08813-y)
Supplement: Supplementary file 2 — Supplementary Material 2. [file 12884_2026_8813_MOESM2_ESM.docx]

**Questionnaire on Breastfeeding Experiences Among Women with Lactation Mastitis**


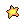
 Dear Participant,

The Department of Breast Surgery at Beijing University of Chinese Medicine Third Affiliated Hospital is conducting a research project related to lactation mastitis. This questionnaire is designed to assess your breastfeeding experiences and related health conditions using a self-developed instrument. The information you provide will be used solely for academic research purposes.

All responses will be kept strictly confidential in accordance with relevant laws and regulations. Only the research team, authorized monitors, the institutional ethics committee, and regulatory authorities (such as the National Medical Products Administration) may access your medical records for research auditing purposes—your personal identity will not be disclosed in any public reports or publications.

Participation in this study is entirely voluntary. You may withdraw at any time without any impact on your medical care or relationship with the investigators. If you agree to participate, please answer all questions truthfully. Your input will help us provide more professional and individualized guidance in future clinical practice.

**If you understand the above content well agree to participate in the study, please sign: [Upload File] ***

*Your signature is required for informed consent for scientific research.*

**Participant demographic and perinatal information**

1. **Your date of birth: [Fill in the blank] ***

_________________________________

1. **Your Ethnicity: [Single Choice] ***

○Han Chinese

○Ethnic minority

1. **Your highest educational level: [Single Choice] ***

○Graduate and above

○Bachelor's degree

○College or below

1. **Are you currently staying at home full-time for breastfeeding: [Single Choice] ***

○Yes

○No, already working

1. **Your body weight: [Fill in the blank] ***

*Please fill in the numbers, default unit: kg. [Example: 58]*

_________________________________

1. **Your height: [Fill in the blank] ***

*Please fill in the number, default unit: cm. [Example: 168].*

_________________________________

1. **Was your current pregnancy planned: [Single Choice] ***

○Yes ○No

1. **How did you conceive this time: [Single Choice] ***

○Natural conception

○Artificial insemination (assisted reproductive technology)

1. **Are you having a singleton pregnancy this time: [Single Choice] ***

○Yes, single pregnancy ○No, twin pregnancy

1. **Is your current delivery your first child: [Single Choice] ***

○Yes ○No

1. **Gestational age of the baby at birth: [Fill in the blank] ***

*Please fill in the combination of numbers, default unit: weeks + days. [Example: 38+3]*

_________________________________

1. **Mode of your current labor: [Single Choice] ***

○Normal labor ○Cesarean section ○Assisted labor (forceps, suction, etc.)

**Medical and obstetric history**

1. **Have you ever been diagnosed with lactation mastitis in the past: [Single Choice] ***

○Yes (Please skip to Question 14) ○No (Please skip to Question 16)

1. **When were you diagnosed with breastfeeding mastitis: [Multiple Choice] ***

□Within 42 days after delivery □42 days to 3 months postpartum

□3 months to 6 months postpartum □6 months to 1 year postpartum

□1 year postpartum

1. **How many times have you been diagnosed with lactation mastitis: [Single Choice] ***

○1～2 times ○More than 3 times

1. **Have you ever been diagnosed with chronic diseases such as hypertension during pregnancy, coronary heart disease, gestational diabetes mellitus, cerebrovascular disease, etc.: [Single Choice] ***

○Yes (Please skip to Question 17) ○No (Please skip to Question 18)

1. **What kind of chronic disease have you been diagnosed with: [Multiple Choice] ***

□High blood pressure during pregnancy □Coronary heart disease

□Gestational diabetes mellitus □Cerebrovascular disease

□Other, please record: _________________*

1. **Do you have a history of breast surgery: [Single Choice] ***

○Yes ○No

1. **Do you have a family history of breast cancer: [Single Choice] ***

○Yes ○No

1. **Do you have a history of drug or food allergy: [Single Choice] ***

○Yes (Please skip to Question 21) ○No (Please skip to Question 23)

1. **What are your allergens: [Multiple Choice] ***

□Ingested allergens, e.g. shrimp, crab, fish, shellfish, beef, lamb, proteins

□Inhalant allergens, such as mold, cockroaches, dogs and cats, dust mites, flowers, grass, trees.

1. **Your allergies are: [Multiple Choice] ***

□Digestive allergy □Allergic dermatitis

□Allergic rhinoconjunctivitis □Allergic asthma

□Other, please record: _________________*

1. **Do you have a history of smoking: [Single Choice] ***

○Yes (Please skip to Question 24) ○No (Please skip to Question 26)

1. **How long have you smoked: [Multiple Choice] ***

□Pre-pregnancy □Pregnancy □When breastfeeding

1. **How long have you been a smoker: [Multiple Choice] ***

□<1 year □1-5 years

□6-10 years □＞10 years

1. **Do you have a history of drinking alcohol: [Single Choice] ***

○Yes (Please skip to Question 27) ○No (Please skip to next Question 29)

1. **How long have you been drinking alcohol: [Multiple Choice] ***

□Before pregnancy □During pregnancy

□While breastfeeding □While breastfeeding

1. **How long did you drink for: [Single Choice] ***

○＜1 year ○1-5 years

○6-10 years ○＞10 years

**Emotional state during breastfeeding and sleep patterns**

1. **Do you feel relaxed when breastfeeding: [Single Choice] ***

○Yes, I feel very relaxed during breastfeeding

○No, I do not feel relaxed during breastfeeding

○I am not currently breastfeeding

1. **Your average length of sleep at night: [Fill in the blank] ***

*Please fill in the numbers, default unit: hours. [Example: 4]*

_________________________________

**Breastfeeding practices and nipple condition**

1. **Are you exclusively breastfeeding: [Single Choice] ***

*Exclusive breastfeeding refers to the feeding of an infant for six months after birth with only the mother's milk, without any other dairy products or milk added.*

○Yes ○No

1. **Do you use a pacifier for your infant: [Single Choice] ***

○Yes ○No

1. **How do you express your milk: [Multiple Choice] ***

□Breastfeeding □Bottle feeding from a breast pump

□Milk collector □No breastfeeding

1. **Do you use a breast pump every day: [Single Choice] ***

○Yes, basically every day ○No, occasionally ○No, do not use breast pump

1. **Have you fed on demand in recent days: [Single Choice] ***

○Yes, I feed the baby when he is hungry or when my breasts are engorged.

○No, feed on time.

1. **Does your baby latch on to your nipple only: [Single Choice] ***

○Yes ○No

1. **Does the baby's latch position cause you pain: [Single Choice] ***

○Yes ○No

1. **Do you have inverted nipples: [Single Choice] ***

○Yes ○No

1. **Do you have short or flat nipples: [Single Choice] ***

○Yes ○No

1. **Do you have cracked nipples, such as bleeding nipples, scabs, ulcers, etc.: [Single Choice] ***

○Yes ○No

**Infant characteristics and feeding behaviors**

1. **Does the infant have intestinal flatulence: [Single Choice] ***

○Yes ○No

1. **Does the infant refuse to breastfeed: [Single Choice] ***

○Yes ○No

1. **Has the baby cried easily in the last few days: [Single Choice] ***

○Yes ○No

1. **Does the infant have difficulty concentrating while breastfeeding: [Single Choice] ***

○Yes ○No

**Breastfeeding Self-Efficacy Short-Form Scale (BSES-SF)**

**This scale describes how confident you feel about your breastfeeding, 1=not at all confident, 2=not very confident, 3=sometimes confident, 4=confident, 5=always confident. Just pick the answer that best expresses your thoughts, there are no right or wrong answers.*

1. **I can always determine that my baby is getting enough milk: [Single Choice] ***

○Not at all confident ○Not very confident ○Sometimes confident

○Confident ○Always confident

1. **I can always successfully cope with breastfeeding like I have with other challenging tasks: [Single Choice] ***

○Not at all confident ○Not very confident ○Sometimes confident

○Confident ○Always confident

1. **I can always breastfeed my baby without using formula as a supplement: [Single Choice] ***

○Not at all confident ○Not very confident ○Sometimes confident

○Confident ○Always confident

1. **I can always ensure that my baby is properly latched on for the whole feeding: [Single Choice] ***

○Not at all confident ○Not very confident ○Sometimes confident

○Confident ○Always confident

1. **I can always manage the breastfeeding situation to my satisfaction: [Single Choice] ***

○Not at all confident ○Not very confident ○Sometimes confident

○Confident ○Always confident

1. **I can always manage to breastfeed even if my baby is crying: [Single Choice] ***

○Not at all confident ○Not very confident ○Sometimes confident

○Confident ○Always confident

1. **I can always keep wanting to breastfeed: [Single Choice] ***

○Not at all confident ○Not very confident ○Sometimes confident

○Confident ○Always confident

1. **I can always comfortably breastfeed with my family members present: [Single Choice] ***

○Not at all confident ○Not very confident ○Sometimes confident

○Confident ○Always confident

1. **I can always be satisfied with my breastfeeding experience: [Single Choice] ***

○Not at all confident ○Not very confident ○Sometimes confident

○Confident ○Always confident

1. **I can always deal with the fact that breastfeeding can be time-consuming: [Single Choice] ***

○Not at all confident ○Not very confident ○Sometimes confident

○Confident ○Always confident

1. **I can always finish feeding my baby on one breast before switching to the other breast: [Single Choice] ***

○Not at all confident ○Not very confident ○Sometimes confident

○Confident ○Always confident

1. **I can always continue to breastfeed my baby for every feeding: [Single Choice] ***

○Not at all confident ○Not very confident ○Sometimes confident

○Confident ○Always confident

1. **I can always manage to keep up with my baby’s breastfeeding demands: [Single Choice] ***

○Not at all confident ○Not very confident ○Sometimes confident

○Confident ○Always confident

1. **I can always tell when my baby is finished breastfeeding: [Single Choice] ***

○Not at all confident ○Not very confident ○Sometimes confident

○Confident ○Always confident

**Edinburgh Postnatal Depression Scale (EDPS)**

**This scale is used to identify the emotional adjustment status of mothers after discharge from hospital, has good reliability and validity, and is widely used abroad. We want to know the answer that best reflects how you have felt over the past 7 days, and there is no right or wrong answer.*

1. **I have been able to laugh and see the funny side of things: [Single Choice] ***

○As much as l always could

○Not quite so much now

○Definitely not so much now

○Not at all

1. **I have looked forward with enjoyment to things: [Single Choice] ***

○As much as l ever did

○Rather less than l used to

○Definitely less than l used to

○Hardly at all

1. **I have blamed myself unnecessarily when things went wrong: [Single Choice] ***

○Yes, most of the time

○Yes, some of the time

○Not very often

○No, never

1. **I have been anxious or worried for no good reason: [Single Choice] ***

○No, not at all

○Hardly ever

○Yes, sometimes

○Yes, very often

1. **I have felt scared or panicky for no very good reason: [Single Choice] ***

○Yes, quite a lot

○Yes, sometimes

○No, not much

○No, not at all

1. **Things have been getting on top of me: [Single Choice] ***

○Yes, most of the time l haven't been able to cope at all

○Yes, sometimes l haven't been coping as well as usual

○No, most of the time l have coped quite well

○No, l have been coping as well as ever

1. **I have been so unhappy that l have had difficulty sleeping: [Single Choice] ***

○Yes, most of the time

○Yes, sometimes

○Not very often

○No, not at all

1. **I have felt sad or miserable: [Single Choice] ***

○Yes, most of the time

○Yes, quite often

○Not very often

○No, not at all

1. **I have been so unhappy that l have been crying: [Single Choice] ***

○Yes, most of the time

○Yes, quite often

○Only occasionally

○No, never

1. **The thought of harming myself has occurred to me: [Single Choice] ***

○Yes, quite often

○Sometimes

○Hardly ever

○Never
